# Prehospital Trauma Care: A Simulation Scenario for Rural-Based Healthcare Providers

**DOI:** 10.7759/cureus.8834

**Published:** 2020-06-25

**Authors:** Robert McCarthy, Bruno Gino, Kerry-Lynn Williams, Philip d'Entremont, Tia S Renouf

**Affiliations:** 1 Family Medicine, Faculty of Medicine, Memorial University of Newfoundland, St. John's, CAN; 2 Emergency Medicine, Madrecor Hospital, Uberlândia, BRA; 3 Pre-Hospital, SIATE - Integrated Trauma and Emergency Assistance System, Uberlândia, BRA; 4 Family Medicine, Memorial University of Newfoundland, Happy Valley-Goose Bay, CAN; 5 General Medicine, University of Limerick, Limerick, IRL; 6 Emergency Medicine, Memorial University of Newfoundland, St. John's, CAN

**Keywords:** prehospital, trauma, simulation, mass casualty, emergency, rural, remote

## Abstract

Trauma is a major cause of premature death and disability worldwide, with a disproportionate number of deaths occurring in rural and remote areas. Prehospital care is a key link in the chain of trauma survival and its role may be currently underestimated. Therefore, addressing deficiencies in prehospital trauma care may help to improve outcomes. Several potential solutions have been proposed to address the disparities that exist in rural prehospital trauma care, some of which focus on educational endeavors. Simulation-based medical education (SBME) is one cost-effective strategy to train healthcare providers in high-acuity, low-opportunity (HALO) scenarios, such as those encountered during major trauma. The aim of this technical report is to present a mass casualty simulation scenario that is intended for healthcare providers in rural and remote locations to refine their skills and comfort level with such cases. It emphasizes prehospital trauma management and effective communication skills among healthcare teams, which are two key elements in improving trauma outcomes.

## Introduction

Trauma is a major cause of premature death and disability worldwide [1,2]. Trauma deaths in rural and remote areas are disproportionately high, and they increase with the degree of rurality [1,3,4]. In Canada, prehospital and in-emergency department (ED) mortality is estimated to be three times higher in rural areas with limited access to trauma centre care [3,4]. Therefore, addressing deficiencies in prehospital management may help improve outcomes for rural trauma victims.

Prehospital models of care

Models for prehospital care vary widely throughout the world. In much of Canada and the United States, patients rely on road transport to reach the nearest hospital that provides emergency medical care. Depending on the location, prehospital care may provide basic lifesaving measures or, when resources are available, more advanced interventions. A significant proportion of Newfoundlanders and Labradorians live rurally, with little access to advanced prehospital care. Resources are often limited at the nearest rural site, and typically a trauma team will be unavailable [1,4]. Personnel at these sites must stabilize patients and transfer them to a tertiary care centre, by road or by air ambulance if weather permits.

Internationally, many areas use road ambulances to varying degrees. One study of emergency medical services (EMS) in several low-middle income countries noted a high degree of variability among the ambulance services that were provided [5]. Most countries included in this study relied on EMS simply as a transportation service, while others employed more advanced medical management in the prehospital setting. Another interesting setting for providing prehospital care is the Royal Flying Doctor Service of Central Australia, which dispatches medically-dedicated aircrafts carrying highly trained personnel and equipment to patients in the Australian outback [6].

In Brazil, prehospital care varies greatly by region, ranging from advanced life support services in developed areas to minimal accessibility to EMS in others [7]. Along major roadways, an intricate system of highway surveillance cameras and inspection vehicles identify motor vehicle accidents (MVAs) 24 hours a day. Highway users may also report accidents. All reporting is centralized at an urban site. Information is then relayed to physicians and nurses located along the highway in ambulances (at about every 80 kilometres). Unfortunately, away from major centres, prehospital care becomes more limited and, in places such as the Amazon, is non-existent [7].

Factors impacting prehospital care

Significant disparities exist regarding rural and remote prehospital care. One common theme in the literature pertains to transportation. It often takes longer for EMS to reach remotely located trauma victims, so their care can be delayed [3,8,9]. Difficult road and sea conditions, as well as harsh weather, can hinder patient retrieval [10]. For undifferentiated trauma patients, faster response and transport times reduce overall mortality [2]. Therefore, addressing ways to improve transportation may improve trauma outcomes for rural patients.

Rural areas are also challenged by limited financial and human resources. EMS attendant availability and level of training vary substantially in lower-income countries [5]. Some countries rely on EMS solely for transport and provide little medical attention in the field [5]. Other nations have more robust EMS, but patients may be reluctant to call them, as they are expensive [5]. Furthermore, rural emergency departments generally have little access to advanced imaging and designated trauma teams [11]. Expected to perform high-acuity low-opportunity (HALO) procedures with limited experience, rural providers need skills training and maintenance. However, since most trained educators are concentrated in academic centres, opportunities are limited for rural-based trainees and practitioners who cannot leave their communities unattended.

Finally, miscommunication between rural physicians and their urban-based colleagues can compromise trauma care [11,12], causing significant delays in transport and the onset of care. This may in turn increase mortality for rural trauma patients.

Improving outcomes in rural trauma prehospital care

A number of potential solutions have been proposed to improve rural trauma patient outcomes [13]. One initiative developed a Rural Trauma Team Development course that focused on effective assessment and resuscitation, along with team communication [13]. This training resulted in reduced transport times, which may translate into improved outcomes for patients.

It is well-established that simulation-based medical education (SBME) is an effective way for healthcare providers in rural areas to learn both procedural skills as well as teamwork and communication skills [9,11,14]. Simulation training can be cost-effective. It can be taught via distance learning methods using telesimulation, allowing urban-based trainers to teach rural learners [11]. This may help build relationships between the two parties, potentially mitigating the miscommunication that can bedevil patient care across the rural-urban divide.

Three-dimensional (3D) printing of anatomical models is an emerging field in SBME, and it provides a feasible and realistic opportunity to practice procedural skills in safe environments [15]. Several studies suggest that participants using 3D-printed models are generally satisfied with the learning experience [15,16]. 3-D printing has also been utilized successfully in trauma SBME [16]. In one study, training with these models was successfully used by rural practitioners who were learning to perform emergent burr holes for traumatic head injuries [16]. Study participants felt that these models were a cost-effective alternative that could potentially fill an identified gap in learning needs for an important HALO procedure, particularly for those healthcare providers located far from large, well-funded neurosurgical programs [16]. Virtual reality (VR) and virtual patients (VP) present another useful way to teach HALO skills, providing realistic, repeated access to situations that are challenging to simulate [17]. This article will describe a mass casualty triage event that may be suitable for VR-based training. 

Effective communication within the healthcare team should not be underestimated, as poor communication can contribute to worse patient outcomes [11,18]. The Situation-Background-Assessment-Response (SBAR) is one example of a closed-loop communication strategy that can be used among team members and for handover during transitions in care to improve outcomes for trauma patients [18]. We have described several educational strategies that may better prepare healthcare providers, especially those in resource-poor, remote locations, to treat patients suffering from major trauma in the prehospital setting.

Herein, we describe a simulation scenario based on an actual case in rural Brazil. The purpose of this simulation scenario is to teach healthcare providers an approach to rural and remote trauma care in the prehospital setting through involvement in a simulated mass casualty case. The learning objectives for this case are:

1. To develop an approach to mass casualty triage 

2. To apply the SBAR approach for standardized communication within the healthcare team

3. To perform a systematic trauma assessment with associated stabilization in the prehospital setting

4. To prepare a patient for safe transport to hospital care

These objectives will be met through formative feedback provided by instructors following the completion of the scenario, including the use of a pre-defined checklist (Appendix A), as well as a didactic session facilitated by the instructors.

## Technical report

Context and inputs

Table 1 lists the context and inputs relating to the simulation scenario.

**Table 1:** Context and inputs

Context
This simulation scenario occurs at the scene of a motor vehicle accident (MVA) along a rural highway, approximately 80 km away from the nearest hospital. The accident involves two vehicles - a van that left the highway, striking a tree near the bank of a river, and a car that is in the woods 500 m away. Inside the car, one victim remains but is unresponsive with three additional victims located within a 10-m radius, who are likely to have been ejected from the car. The van contains two victims, both restrained with neither showing signs of movement. The scenario begins with the arrival of the healthcare team on the scene of the MVA. One to two learners (rural-based physicians or residents) will assume the roles of physicians and begin directing the team in the management of the case
Inputs
Personnel:
Two learners (residents or rural-based physicians)
Four confederates playing the roles of two nurses and two paramedics
The roles of one nurse and one paramedic may be played by additional learners if available
Two instructors (emergency room physicians)
Six standardized patients (SPs) playing the victims
Set-up:
Available materials (as outlined in Table 2)
Optional: locally available task trainers for procedural skills
Airway management
Chest tube insertion

As mentioned above, the scenario is designed to accommodate two learners who assume the roles of physicians arriving at the scene by ambulance. Each ambulance also contains a nurse and a paramedic. At least one nurse and one paramedic should be played by confederates who are trained to provide appropriate prompts, as outlined in Table 3 below. The roles of one nurse and one paramedic may be assumed by additional learners if available or, alternatively, may also be played by confederates.

Table 2 outlines the equipment and medications that are available to the healthcare providers at the scene of the accident. This equipment does not need to be readily available to run the simulation; however, learners should be made aware of what the limitations are. For example, two ambulances are not necessary to run the simulation; however, learners should be aware that they have the capacity to transport a maximum of four victims (two per ambulance) at one time.

**Table 2:** Resources available to learners OPA: oropharyngeal airway; BVM: bag valve mask; PEEP: positive end-expiratory pressure; ET: endotracheal; IV: intravenous

Equipment	Medications	Fluids
Two ambulances	Xylocaine 2% without epinephrine	Normal Saline (0.9%)
Four stretchers (two per ambulance)	Xylocaine 2% with epinephrine	Ringer’s Lactate
Four immobilization boards	Epinephrine injection (1:1000)	Dextrose 5% (D5W)
Oxygen capabilities (oxygen cylinders with valves, pressure gauge and flowmeter with mask)	Procainamide (100 mg/ml)	Mannitol 25%
Cardioverter with non-invasive external pacing capabilities and disposable electrodes	Dopamine (40 mg/ml)	Distilled Water
Infusion pumps	Isoproterenol (0.2 mg/ml)	Calcium gluconate 10%
Emergency kit containing: stethoscope, OPAs of various sizes, disposable gloves, sterile surgical and gauze dressings, cervical spine collars, splints for immobilization, reflective vests, flashlights, personal protective equipment, BVM with masks of various sizes and PEEP valve	Atropine (0.4 mg/ml)	Magnesium sulfate 50%
Airway kit containing: ET tubes of various sizes, tracheal aspiration probes of various sizes, procedure gloves, xylocaine jelly, infant laryngoscope blades (0, 1), adult laryngoscope, curved blades (1, 2, 3), elastic bougie, disposable scalpel	Hydrocortisone (Solu-Cortef®) (50 mg/ml)	Sodium bicarbonate 8.4%
Venous access kit: procedural gloves, sharps container, tape, IV catheters of various sizes, tourniquet, scissors, antiseptic gauze wipes, chest tube kit, procedural gloves, antiseptic solution, selection of needles (16-24 G) and syringes, xylocaine 2% solution, suture kit (including suture material, suture scissors, and needle driver), scalpel, curved clamp (for blunt dissection), bottles for chest drainage, chest tube (with clamps), gauze and dressings of various sizes	Phenytoin (50 mg/ml)	
	Morphine (4 mg/ml)	
	Diazepam (5 mg/ml)	
	Haloperidol (5 mg/ml)	
	Chlorpromazine (25 mg/ml)	
	Dimenhydrinate (50 mg/ml)	
	Metoclopramide (5 mg/ml)	

The roles of instructors should be assumed by two emergency medicine physicians. One should be designated as a facilitator to oversee the running of the simulation, answering any questions that arise from the participants. A second instructor should be present to assess individual procedural skills, communication, and the overall organization of the case, using the associated checklist to guide the assessment (Appendix A). It is recommended that instructors run through the scenario beforehand to identify any technical issues or limitations with the running of the simulation.

Process

This is a hybrid simulation scenario, using standardized patients (SPs) playing the roles of victims and confederates who play the roles of other team members. In this particular scenario, the team includes a physician, a paramedic, and a nurse in the first ambulance on the scene. An additional ambulance occupied by another nurse, a paramedic, and a physician may be called for additional support. If there is a limited supply of confederates, additional learners may also assume the roles of other team members. The confederates who assume the roles of the nurse(s) and paramedic(s) will be responsible for providing learners with the appropriate information when it is requested, including vital signs and other pertinent physical exam findings. When a participating learner acknowledges that a procedure may need to be performed, mannequins and 3D-printed models could be utilized to demonstrate procedural skills, such as airway management with oropharyngeal airway (OPA) insertion, bag-mask ventilation (BMV), and endotracheal intubation (ETI), as well as chest tube insertion.

Prior to the beginning of the scenario, learners are introduced to the fictional contract, which requires acknowledging that although the simulated case is fictional, they are to treat it as if it is “real”, acting as they would in clinical practice so as to make the simulation worthwhile. Learners should be advised that the simulation is purely formative. During this time, the instructors for the scenario should be introduced, along with any confederates and their defined roles (i.e. paramedic, transport nurse, etc). The procedure will be outlined for learners to obtain necessary information, such as updated vitals and physical exam findings. Limitations of the scenario, such as investigations that will not be available (i.e. labs, imaging) along with limits to the various task trainers should be discussed. Finally, learners will be encouraged to assign roles in preparation for the beginning of the scenario. The scenario will terminate when patients have been adequately stabilized and prepared for transfer, with learners “calling ahead” to provide handover to the accepting facility. Instructors should be appointed ahead of the simulation and should be emergency medicine physicians. Table 3 is a detailed, step-wise scenario template that was developed and should be provided to the facilitators and simulation laboratory support staff about 30 days prior to the scenario so that they could brief the SPs and gather any necessary supplies. Following the completion of the simulation scenario, facilitators and learners will participate in a debriefing and didactic session, as outlined below. As part of the debriefing process, formative feedback will be provided to learners.

**Table 3:** Simulation scenario design PPE: personal protective equipment; EMS: emergency medical services; GCS: Glasgow Coma Score; A&O: alert and oriented; ABC: Airway, Breathing, Circulation; T: temperature; BP: blood pressure; HR: heart rate; SpO2: oxygen saturation; BVM: bag valve mask; OPA: oropharyngeal airway; SBAR: Situation, Background, Assessment, Response

Pre-scenario			
You are a part of a mobile trauma response unit near Uberaba, Brazil, along with a nurse and paramedic. It is 8:00 am at the start of your Friday shift. It rained heavily the night before, but it has cleared this morning. You get a call saying there was a car accident involving two vehicles about 80 km up the road. There is a van on the bank of the river with signs of a high-energy collision with a tree. There are two restrained victims inside the van who are not moving. The other car is in the woods about 500 m from the van. A victim is inside with no body movements. Three additional victims who were seemingly ejected from the car are within a 10-m radius. The ambulance that is assigned to your team can accommodate up to two victims. Another ambulance, with a physician, nurse, and paramedic, is currently nearby and may be called if further assistance is needed			
State	Status	Learner actions	Operator notes
1. Arrival and scene survey		- Call for more help	Modifiers:
		- Get appropriate PPE/equipment	- If no mention of traffic/scene safety, EMS prompt
		- Assess scene safety	
		- Begin to triage casualties	
2. Casualty identification and triage	Van 1: driver, 60-year-old male, seat belt fastened, open chest trauma, no signs of life (victim A)	- Triage code black	Triggers:
	Van 1: passenger 1, 70-year-old female, seat belt fastened, head rotated 180 degrees, no signs of life (victim B)	- Triage code black	All casualties triaged → 3. Stabilization (red)
	Car 2: driver, 35-year-old male, seat belt fastened, cervical spine exposed, no sign of life (victim D)	- Triage code black	
	Car 2: ejected victim 1, 25-year-old female, supine, GCS 4, asymmetric chest rise (victim A)	- Triage code red	
	Car 2: ejected victim 2, 25-year-old female, A&O x3, GCS 15, complaining of severe right leg pain (victim B)	- Triage code green	
	Car 2: ejected victim 3, 30-year old-male, initially conscious and verbalizing, complaining of severe pain in the right chest (victim C)	- Triage code yellow	
3. Stabilization (red)	T 35.5 °C	- Assess ABCs	Modifiers:
	HR 130	- Protect C-spine	- BVM → SpO2 increases to 78%
	BP 60/30	- Insert OPA and start bagging with 100% oxygen	- No comment on asymmetric chest rise → patient continues to deteriorate
	SpO2 70% on room air	- Obtain IV access, start fluid bolus	- Bolus → BP increases to 70/40, HR 120
	GCS 4 (extends to pain)	- Slightly asymmetrical chest rise (worse on right) noted	- Chest tube inserted → 1.7 L blood drained. → BP 90/70, HR 100, SpO2 97%
	Smells of alcohol	- Chest tube inserted on right	Triggers:
		- Secondary survey, immobilize and warm	Immobilization → 4. Stabilization (yellow)
4. Stabilization (yellow)	T 36.4 °C	- Assess ABCs	Modifiers:
	HR 140	- Protect C-spine	- O2 by non-rebreather → SpO2 stays at 90%
	BP 100/60	- Apply 100% O2 by non-rebreather mask	- During assessment, if no recognition of tracheal deviation → patient continues to deteriorate, SpO2 decreases to 50%, BP decreases to 45/20
	SpO2 90% on room air	- Obtain IV access	- Needle decompression → BP 100/50, HR 95, SpO2 93%
	GCS 15	- Recognize tracheal deviation and needle decompress (or insert chest tube)	Triggers:
	Confused and verbalizing, complaining of pain to the right chest	- Secondary survey and immobilize, and warm	- Immobilization → 5. Stabilization (green)
5. Stabilization (green)	T 35.4 °C	- ABCs	Modifiers:
	HR 140	- Protect C-spine	- If foot not noticed → patient to cry out in pain
	BP 110/70	- Obtain IV access	- Bleeding controlled → HR 130
	SpO2 98% on room air	- Primary survey – traumatic amputation of right foot; crepitus in the right thigh	- Leg splinted → HR 120
	GCS 15	- Apply pressure to wound, consider tourniquet if bleeding continues	
	A&O, denies neck pain, complaining of severe right thigh/leg pain	- Splint leg	Triggers:
		- Fluid bolus	- Immobilization → 6. Transport
		- Secondary survey and immobilization	
6. Transport	As above	- Call with reports on each patient (SBAR)	Triggers:
		- Consider human resource allocation (physician to go with most unstable casualty)	- If no report → prompt by nurse for same
			- SBAR → end of scenario

The above simulation scenario includes key elements of prehospital trauma care in a rural setting. Therefore, it is intended to be used as a learning tool for rural-based healthcare providers, mainly medical trainees and physicians who often have limited exposure to prehospital trauma management.

Debriefing

Following the scenario, all learners should be given the opportunity to debrief, highlighting whether their overall learning experience was positive or negative and to discuss what they had learned during the simulation. During this time, they are also encouraged to ask questions and to provide feedback that may help to improve their own knowledge, as well as future runnings of the simulation. Debriefing will be overseen by the two instructors of the simulation. We encourage advocacy-inquiry as an appropriate model for the debrief, allowing facilitators the opportunity to provide constructive feedback and subsequently allowing learners to reflect on and discuss their individual experiences throughout the simulation.

Following the debrief, approximately 30 minutes of didactic instruction is included to provide additional teaching around the learning objectives of the case. The inclusion of a didactic session immediately following the simulation has been shown to consolidate key clinical information and to highlight knowledge gaps for further learning. [9] Demonstrations of the relevant procedural skills, such as BMV, OPA insertion and chest tube placement can also be reviewed using any available mannequins and task trainers. The main teaching points to be covered in the didactic session are summarized in Table 4**.**

**Table 4:** Teaching points for the didactic session GCS: Glasgow Coma Score; OPA: oropharyngeal airway; BMV: bag-mask ventilation; C-spine: cervical spine; O2: oxygen; NRB: non-rebreather; BP: blood pressure; HR: heart rate; ABCs: Airway, Breathing, Circulation; DRE: digital rectal exam; SBAR: Situation, Background, Assessment, Response; ABCDE: Airway, Breathing, Circulation, Deformity, Exposure

Learning objective	Teaching points
To develop an approach to mass casualty triage	When encountered with a mass casualty trauma scenario, such as in our case, emphasis is placed on maximizing the number of lives saved. A colored tagging system has been developed to quickly prioritize efforts towards victims who may be saved, given the immediate availability of resources as well as the extent of a victim’s injuries. The colored tagging system is summarized in Table 5. In our particular case, our immediate efforts should have been targeted towards the three victims in the car with signs of life. Victim A, who was most vitally unstable with a GCS of 4, should have received a red tag, indicating a need for immediate treatment. Victim C, who verbalized severe right-sided chest pain but also had unstable vitals, should have been the next priority and thus received a yellow tag. Victim B, with a GCS of 15 and leg pain, should have received a green tag, as his injuries do not appear to be immediately life-threatening. Victim D in the car, as well as both victims in the van, should have received black tags, as their injuries were not amenable to the level of care that may be provided in the field [19]
To apply the SBAR approach and closed-loop communication strategies for standardized communication within the team	The importance of effective communication in complex medical scenarios should not be underestimated. Developing a systematic approach to communication within a healthcare team is essential to the prevention of medical errors [18]. Closed-loop communication, in which the receiver of information confirms what they hear to the sender who then affirms or corrects the message, is one effective way to mitigate error within complex team-based scenarios [18]. During critical events, specifically, when many essential tasks happen simultaneously, closed-loop communication helps to ensure that patient care remains organized. Another point of communication that is often a source of medical error is the handover, when a patient’s care is assumed by another team or new members join an existing team. One systematic approach for handover is the SBAR technique, which refers to Situation, Background, Assessment, and Response [18]. Using the SBAR approach helps to ensure that the necessary background information (including the care received up to the point of handover) along with the team’s thought process is clearly communicated in the handover period so that the transition has less impact on a patient’s care. Handovers present a good opportunity for rural-based physicians to discuss with urban colleagues the context in which they are working. This may help mitigate the aforementioned misunderstanding that often occurs between urban- and rural-based providers
To perform a systematic trauma assessment with associated stabilization in a prehospital setting	A systematic approach to trauma assessment first involves attending to ABCs (Airway, Breathing, and Circulation). In our particular scenario, victim A (who received a red tag) is the first patient to be treated. This victim’s physical examination findings (GCS 4, asymmetric chest rise, hypotension, tachycardia, oxygen saturation of 70%) indicate a need for immediate assessment and management. In this particular case, her altered level of consciousness and unstable vitals warrant the management of her airway, initially with an OPA and BMV. Special consideration should be given to C-Spine stabilization in all trauma patients who require airway interventions. Given her asymmetric chest rise and hypotension in the context of trauma, we would be suspicious of a hemothorax or large pneumothorax under tension. At this point, a chest tube could be inserted, which results in an improvement in the patient’s vitals. OPA, BMV, and chest tube insertion could be demonstrated at this time using any available task trainers or mannequins. Victim B, who initially received a yellow tag, was mentating well (GCS 15) and was verbal, thereby protecting his own airway. However, his physical exam findings of tracheal deviation, O2 saturation of 90% despite the use of 100% O2 via NRB mask and BP of 100/50 are concerning. In the context of trauma, this constellation of findings may be amenable to needle decompression. This skill could be demonstrated by instructors at this time. Finally, victim C was given a green tag, as he was mentating well and crying out in pain, indicating a protected airway. Although his physical exam findings were also concerning, with a BP of 110/70 and an HR of 140, he was in the least need of immediate attention. When the higher priority victims are stabilized, attention should have been paid to stabilizing victim C’s vitals, which were likely abnormal secondary to hemorrhage in the context of trauma. By reducing and immobilizing the fracture and using a tourniquet or pressure to limit his blood loss, his vitals improved. An IV fluid bolus to replace his lost volume also helps to stabilize his vitals. For all victims of trauma, IV access and C-Spine evaluation are vital to their immediate care. Following the stabilization of each patient’s ABCs, attention should be focused on disability, which highlights the importance of performing a neurological exam (looking for signs of herniation or intracranial bleeding). Finally, all patients should have all clothing removed and they should be inspected for other areas of injury. A DRE to assess for rectal tone rounds out the primary neurological assessment. Emphasis should then be placed on exposure and the environment, ensuring that patients remain warm and dry, given the propensity for patients with trauma to experience hypothermia. This completes the primary survey for each victim
To prepare a patient for safe transport to hospital care	In order to expedite patient care on arrival, the nearest center should be notified by a healthcare provider en route. Again, SBAR may be an appropriate way to communicate with accepting staff members in this type of scenario. Patients should be frequently re-assessed en route to the facility, with a particular emphasis placed on the ABCDEs. As previously mentioned, C-Spine evaluation and possible immobilization is essential. Endotracheal intubation may be required prior to road transfer for victim A. Rapid sequence intubation may be demonstrated for learners to conclude the didactic component

 Table 5 summarizes the principles of mass casualty triage.

**Table 5:** Principles of mass casualty triage

Tag color	Triage	Assessment	Disposition
Red	Highest priority; these patients have life-threatening injuries	Spontaneously breathing when airway-opening maneuvers performed; respiratory rate >30/min; radial pulse absent; capillary refill >2 sec; unable to follow verbal commands	Immediate attention required
Yellow	Second priority; these patients may have life-threatening injuries but are not imminently deteriorating	Spontaneously breathing; respiratory rate <30/min; radial pulse present; capillary refill <2 sec; obeys verbal commands	Care may be delayed for now. Status not expected to deteriorate over several hours
Green	Third priority; these patients have non-life-threatening injuries	Able to walk and remove themselves from the scene (“walking wounded”)	Care may be delayed as status is not likely to deteriorate
Black	Lowest priority; these patients are unlikely to survive given the severity of the injuries and/or the level of care available	Not spontaneously breathing despite attempts to position the airway	Provide analgesia and other palliative measures

## Discussion

This scenario is designed to teach the principles of trauma management and mass casualty triage in the prehospital setting. It has been tailored to simulate rural and remote regions where access to prehospital care and resources is often limited. The provision of appropriate prehospital care can have a significant impact on trauma-associated mortality [1]. SBME provides an opportunity for healthcare providers to improve their knowledge and skills, becoming more comfortable with considerations of rural and remote medicine, and prehospital care [9,11,14]. This may help them to feel more comfortable with the management of trauma in areas with limited resource availability.

Our scenario also places an emphasis on communication, as deficiencies here can significantly impact care [11,12]. Firstly, for learners working in rural and remote regions, the practice of closed-loop communication throughout the simulation, as well as the handover of patients, are crucial skills to obtain in clinical practice. Additionally, urban-based providers who participate can gain an appreciation for the key differences in resource availability when located outside of tertiary centres, thereby addressing the contextual misunderstanding that often hinders communication between rural- and urban-based healthcare practitioners.

## Conclusions

Major trauma is a significant cause of premature death and disability worldwide. The provision of patient care in the prehospital setting is pivotal in mitigating these outcomes. In rural settings, trauma-associated mortality is significantly higher than in urban areas. Therefore, addressing ways to improve prehospital care, particularly in rural and remote settings, may help to improve trauma outcomes in these regions. The aforementioned simulation offers rural healthcare providers an opportunity to practice their skills in mass casualty triage scenarios, an important HALO situation in a rural or remote context. Through participation in this simulation, we hope that providers will gain confidence and skills that will improve their ability to appropriately manage these situations when they arise.

## Appendices

**Table 6:** Appendix A - formative evaluation tool

	Yes	No
Objective 1: mass casualty triage		
Assesses scene		
Wears appropriate personal protective equipment		
Asks for additional help		
Acknowledges limited resources and need for triage		
Asks facilitator for condition and vital signs for all victims		
Appropriately assigns triage tags as follows:		
Van - victim A (black)		
Van - victim B (black)		
Car - victim A (red)		
Car - victim B (green)		
Car - victim C (yellow)		
Car - victim D (black)		
Allocates available resources appropriately (i.e. attends to victims in the following order: red→ yellow → green → black)		
Objective 2: effective team communication		
Encourages closed-loop communication of all team members		
Delegates tasks appropriately to other team members		
Appropriately uses the SBAR (Situation, Background, Assessment, Response) approach when communicating with accepting providers		
Objective 3: to perform a systematic trauma assessment with associated stabilization in a prehospital setting		
Car victim A (red tag):		
Reassesses vitals		
Assesses airway		
Acknowledges need for intervention		
Selects appropriately-sized oropharyngeal airway (may demonstrate on mannequin)		
If demonstrated, uses appropriate technique.		
Bag-mask ventilation (may demonstrate on mannequin)		
If demonstrated, uses appropriate technique.		
Maintains C-Spine immobilization throughout airway intervention		
Assesses breathing (auscultation) *prompt: reduced air entry to right lung		
Acknowledges potential causes (hemothorax, pneumothorax)		
Suggests chest tube Insertion (may demonstrate with task trainer)		
If demonstrated, uses appropriate technique		
Obtains IV access x 2 (large-bore)		
Administers fluid bolus		
Reassesses vitals		
Immobilizes patient in preparation for transport		
Allocates team member to continue monitoring patient		
Car victim B (yellow tag):		
Reassesses vitals		
Airway assessment (patent)		
Assesses breathing		
When provided with physical findings, suggests potential causes for victim’s problem (including tension pneumothorax)		
Suggests needle thoracostomy (may demonstrate on mannequin if available)		
If available: demonstrates appropriate technique		
Obtains IV access x 2 (large bore)		
Administers fluid bolus		
Reassesses vitals		
Immobilizes patient in preparation for transport		
Allocates team member to continue monitoring patient		
Car victim C (Green)		
Reassesses vitals		
Assesses airway (patent)		
Assesses breathing		
Assesses circulation		
Obtains IV access x 2 (large bore)		
Administers fluid bolus		
Acknowledges that fracture likely contributing to tachycardia/hypotension		
Suggests reduction of fracture		
Suggests tourniquet or direct pressure		
Reassesses vitals		
Immobilizes patient in preparation for transport		
Re-assessment of car victims A-C		
Reassesses vitals		
Ensures adequate exposure		
Neurological exam, including digital rectal exam for rectal tone		
Ensures patients are appropriately covered to maintain normothermia		
Objective 4: to prepare a patient for safe transport to hospital care		
Acknowledges the need to call ahead to the accepting facility		
Calls ahead and provides appropriate information		
Appropriately prioritizes transport of sickest patient first (red tag)		
